# Post Learning Sleep Improves Cognitive-Emotional Decision-Making: Evidence for a ‘Deck B Sleep Effect’ in the Iowa Gambling Task

**DOI:** 10.1371/journal.pone.0112056

**Published:** 2014-11-19

**Authors:** Corrine J. Seeley, Richard J. Beninger, Carlyle T. Smith

**Affiliations:** 1 Centre for Neuroscience, Queen's University, Kingston, Ontario, Canada; 2 Department of Psychology, Queen's University, Kingston, Ontario, Canada; 3 Department of Psychology, Trent University, Peterborough, Ontario, Canada; Harvard Medical School, United States of America

## Abstract

The Iowa Gambling Task (IGT) is widely used to assess real life decision-making impairment in a wide variety of clinical populations. Our study evaluated how IGT learning occurs across two sessions, and whether a period of intervening sleep between sessions can enhance learning. Furthermore, we investigate whether pre-sleep learning is necessary for this improvement. A 200-trial version of the IGT was administered at two sessions separated by wake, sleep or sleep and wake (time-of-day control). Participants were categorized as learners and non-learners based on initial performance in session one. In session one, participants initially preferred the high-frequency reward decks B and D, however, a subset of learners decreased choice from negative expected value ‘bad’ deck B and increased choices towards with a positive expected value ‘good’ decks (decks C and D). The learners who had a period of sleep (sleep and sleep/wake control conditions) between sessions showed significantly larger reduction in choices from deck B and increase in choices from good decks compared to learners that had intervening wake. Our results are the first to show that post-learning sleep can improve performance on a complex decision-making task such as the IGT. These results provide new insights into IGT learning and have important implications for understanding the neural mechanisms of “sleeping on” a decision.

## Introduction

Decision-making can be difficult. To make a decision, individuals must use current knowledge to predict and weigh all potential options. The decision process is particularly difficult when options are complex and involve multiple risks and benefits, such as the decision to take a new job or move to a new city. A common word of advice to individuals before making an important or difficult decision is to ‘sleep on it’. This implies that when weighing the risks and benefits of multiple options, sleep may help sort through information to provide clear insight to the answer. Although it is a common practice, there has yet to be strong evidence that sleep facilitates the decision-making process.

The Iowa Gambling Task (IGT) is a widely used task that was designed to mimic real life decision-making. In the IGT, individuals experience rewards and punishments as they select from four card decks: A, B, C, D. Deck A and B are ‘bad decks’ that have high immediate rewards ($100 per draw) and larger comparative punishments. Deck C and D are ‘good decks’ that have relatively smaller immediate rewards ($50 per draw) but smaller comparative punishments. The good and bad decks result in a $250 positive or negative expected value (EV), respectively, per 10 card selections [1]. Recent studies suggest that initial deck preferences are driven towards decks with a high probability (0.9) of reward (bad deck B and good deck D) compared to decks with a lower probability (0.5) of reward (bad deck A and good deck C) [2], [3], [4]. Thus, advantageous decision-making relies on shifting choice away from bad deck B and replacing choices with good decks C and/or D [5], [6].

A wide body of literature has identified the neural mechanisms underlying learning of the IGT. Areas linked to emotional processing, including the ventral striatum, insula and amygdala have heightened activity during task acquisition [7], [8], [9]. These areas are thought to communicate affective information to the ventral medial prefrontal cortex (vmPFC) which integrates previous emotion to reward and loss to guide future choice [10], [11], [12]. Over time, associations are stored in long-term memory via connections to hippocampal memory systems [13].

Recent evidence suggests that the neural circuitry underlying IGT learning is sensitive to periods of sleep deprivation [14]. Killgore et al. [15] revealed that compared to well-rested controls, 46 hours of sleep deprivation can lead to decision-impairments in the IGT, marked by increased choice toward bad decks combined. Currently, it has yet to be investigated whether learning in this system can be enhanced across multiple sessions, and across periods with intervening sleep. In general, a period of sleep following learning promotes synaptic changes and strengthens memories of recently acquired information [16], [17], [18]. Recent work suggests that post-learning sleep helps facilitate insight into complex strategies and rule-based learning [19], [20], [21], [22]. Furthermore, it is well known that sleep enhances memories for emotionally relevant stimuli [23], [24]. Considering the IGT is a decision-making task that integrates complex cognitive and emotional information, and is sensitive to the effects of sleep deprivation, we suggest that post-learning sleep may facilitate this unique process.

A recent study by Pace-Schott et al. [25] reported that individuals who engaged in sleep following 100-trials of the original IGT had a significant improvement in choices from positive EV decks compared to individuals that had intervening wakefulness. However, because the sleep and wake group had differences in time of re-testing (morning and evening) and hours of wakefulness prior to retesting (0–1 and 12 hours), conclusions could not be made on whether post-learning sleep enhanced IGT decision making. The authors suggest that future research should control for potential influences of time-of-day and amount of prior wakefulness on IGT performance. A second study by Abe et al. [26] reported sleep-dependent improvement using an instructional version of the IGT. Prior to engaging in sleep or wake, individuals were instructed to click on each deck 6 times in a predetermined order. The authors report sleep-dependent improvement, however, recent studies have revealed that instructed feedback of the IGT does not engage the same underlying decision processes (i.e., activation of vmPFC) as the original version [7], [8]. Given this, it is unlikely the instructed version of the task captured the decision processes of the original IGT.

The goal of our study was to determine whether post-learning sleep enhances performance on the IGT. To do this, we administered the IGT and retested participants after 12 hours of wakefulness, 12 hours with intervening sleep, or 12 hours with intervening sleep followed by 12 hours of wakefulness. The latter group was used to investigate potential influences of time-of-day and hours of wakefulness before retesting. To measure improvement, we used the traditional method of analysis (positive EV decks combined) reported by Pace-Schott et al. [25] and Abe et al. [26]. Additionally, we expanded the analysis by investigating improvement across individual decks (A, B, C, D).

The major questions addressed were: 1) are deck choices initially driven toward decks with high reward frequency, 2) how do individual deck choices shift as learning occurs, and 3) does intervening sleep between sessions enhance performance? Given previous work, we hypothesized that there would be an initial preference for deck B and D [2], [3], [4] and that learning would reflect a decrease in choices from bad deck B and increase in choices from good decks C and/or D [5], [6]. We expected that those who engaged in post-learning sleep would show greater overall improvement than those in the post-learning wake condition.

## Methods

### Pilot Task: Task Development

Within the sleep literature, sleep-related improvement is largely reliant on initial insight being achieved prior to sleep [19], [27], [28], [29], however, effects can be lost if participants hit a ceiling during initial learning [19], [30]. Given that very few studies have administered the IGT across multiple sessions, our initial goal was to identify a version where individuals show initial improvement during session one, and have room for improvement during session two. We piloted two versions of the task, the original version with good and bad decks placed side by side (A, B, C, D), and a more difficult shuffled version [12], [31] with good and bad decks separated (C, A, D, B). Pilot testing of the original version revealed a ceiling effect within the first 100 trials, with no room for improvement in a second session. Pilot testing of the 200-trial shuffled version revealed a moderate improvement in session one and further improvement during session two. For this reason, we chose the 200-trial shuffled version for our study. This version maintained the same punishment and reward structure [1] and shift in deck choice across learning as the original version.

### Participants

Participants were recruited through poster advertisements on Trent University and Queen's University campuses. Prior to selection, they were administered a telephone and online screening questionnaire. The screening measures confirmed participants had healthy sleep hygiene. This included being free from atypical sleep patterns (sleep time outside 22:00–09:00, shift work or napping), sleep disorders, use of sleep altering medications, head injuries, history of depression, physical ailments, excessive alcohol use (>10 drinks per week), excessive caffeine use (>4 caffeinated beverages per day) and nicotine use. It also confirmed that participants maintained a healthy sleep schedule, going to bed between 22:00–24:00 and waking 07:00–09:00 daily, with no trouble sleeping and experiencing little to no sleep disruptions throughout the night. This sleep schedule and sleep hygiene behaviour were then used for guidelines for participants to follow during participation. Participants that met these criteria were randomly assigned to a 12-hour sleep condition (n = 33; female  = 28; mean age  = 20.6± SEM 0.37 years), 12-hour wake condition (n = 26; female  = 21; mean age  = 20.1± SEM 0.41 years), or 24-hour sleep/wake control condition (n = 33; female  = 27; mean age  = 20.3± SEM 0.38 years) prior to participation.

### Protocol

**Figure 1:**
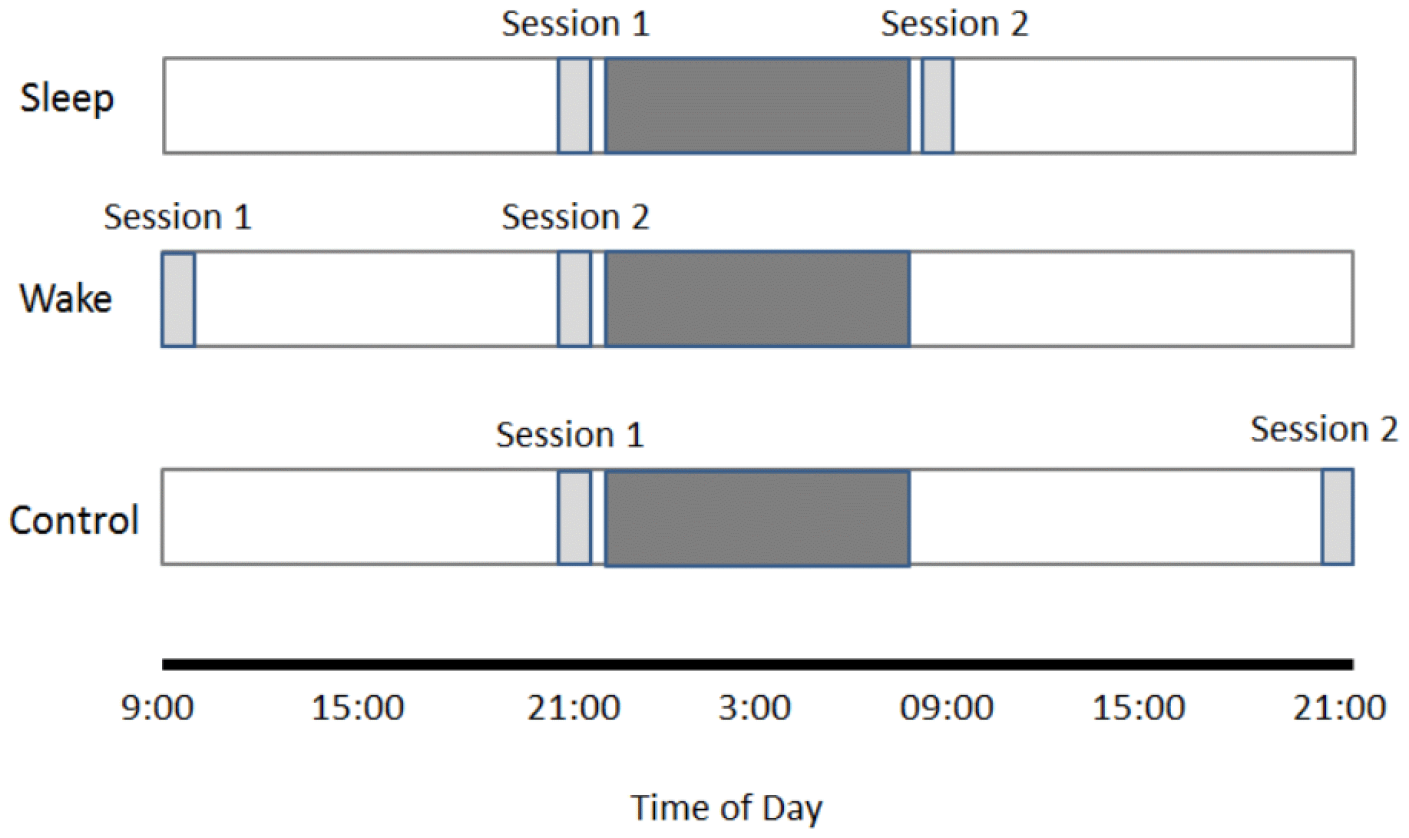
Experimental Design. The experimental design for participants in the sleep (n = 33), wake (n = 26) and sleep/wake control (n = 33) conditions. The shaded bar indicates the period of time in which sleep occurs.

Participants visited the lab one week prior to participation where they filled out a written consent form and were given an Actiwatch and sleep and activity diary. They were reminded to adhere to the sleep guidelines for the week prior to participation, as well as prior to and between testing sessions. They were also instructed to avoid caffeine use after 15:00, refrain from using alcohol on days with scheduled sessions, and to avoid caffeine use prior to morning testing sessions. One week later, participants returned to the lab at two separate sessions to perform our 200-trial version of the IGT. The task was administered during session one, with session two after 12 hours with intervening sleep (21:00–22:00 and 09:00–10:00), 12 hours with intervening wake (09:00–10:00 and 21:00–22:00) or 24 hours with intervening sleep and wake (sleep/wake control; 21:00–22:00 and 21:00–22:00) (Fig. 1).

To ensure participants were well-rested, and to investigate whether self-reported sleepiness levels changed across sessions and between conditions, the Epworth Sleepiness Scale (ESS) was filled out at the beginning of each session [32]. Verbal instructions were given, as previously reported in Seeley et al. [33] and the task was administered for 200 trials, after which the participant was told the task was over. The task began with $2000 in virtual money. As they selected from decks, the reward and punishment value, net total and their updated cumulative total was displayed on the screen. Decks were recycled after 40 trials, allowing for unlimited choice from each deck. The same procedure was followed in session two. After session two participants returned the Actiwatch, were debriefed and reimbursed $35 for their time. This study received approval from the Queen's University General Research Ethics Board and Trent University Research Ethics Board. Because participants slept at home and could not be directly monitored, actigraphy (Actiwatch, Mini Mitter, Inc, Bend OR) and self-report diaries were used to confirm that participants adhered to the guidelines, were well-rested, and had similar sleep histories.

### Actigraphy Measures and Epworth Sleepiness Scores

To evaluate participants' sleep history prior to participation, one-way simple effects ANOVA of condition (sleep, wake, sleep/wake) were performed with average total sleep time across the week (average TST; minutes) and total sleep time prior to session one (prior TST; minutes) as dependent variables. Results revealed a non-significant effect on average TST (F[2, 85]  = 0.1, p = 0.99), and prior TST (F[2, 85]  = 0.1, p = 0.99). The sleep, wake and sleep/wake conditions were similarly well-rested and had a similar average TST ±SEM (467.8±8.7, 469.2±7.6 and 467.5±7.4) and prior TST (465.1±13.0, 463.0±14.8, and 462.5±21.6), respectively. Furthermore an independent sample t-test revealed that minutes of total sleep time between session one and session two were similar in the sleep (431.5±89.9) and sleep/wake control (457.2±55.7) (t[61]  = −1.4, p = 0.17).

To investigate whether self-reported sleepiness differed between sessions and across conditions a two-way repeated measures ANOVA with condition (sleep, wake, sleep/wake) and session (1 and 2) as independent variables were performed with ESS score as a dependent variable. The condition x session ANOVA revealed a non-significant interaction for ESS scores (F[2, 85]  = 1.5, p = 0.23). The ESS scores (± SEM) in session one were 6.0 (±0.58), 6.3 (±0.54), and 6.4 (±0.51) and for session two were 6.7 (±0.83), 5.2 (±0.79), and 5.7 (±0.73) in the wake, sleep, and sleep/wake control conditions, respectively. All scores were well below the criteria that would indicate daytime sleepiness [32] and did not change across sessions. Overall, the actigraphy data and ESS scores verified that participants were similarly well-rested throughout the entire duration of the study.

### Statistical Analysis

#### Session One: Initial Learning

ur initial questions of interest were: 1) Are the initial deck preferences driven toward deck B and D? and 2) As learning occurs, how do individual deck choices shift? We also aimed to identify potential differences in deck preferences among conditions (sleep, wake, sleep/wake) to ensure there were no group differences in session one. The 200-trials from session one were split into 4, 50-trial blocks [34]. The dependent variable was proportion of choices (total draws chosen/total number of possible draws) from each deck for each condition within each block. To investigate how deck preferences changed from block 1 to block 4, and to evaluate possible differences among conditions we performed a three-way analysis of variance (ANOVA) with independent variables of block (1, 2, 3, 4), deck (A,B,C,D), and condition (sleep, wake, sleep/wake). Greenhouse-Geisser corrections were made to avoid the effects of sphericity violations. To investigate initial and final deck preferences a one-way simple effects ANOVA of decks was performed for block 1 and block 4 separately, followed by Tukey tests for pairwise comparisons. In addition, to investigate how deck choices shifted, a repeated measures ANOVA of block (1, 2, 3, 4) was performed for each deck followed by paired t-tests comparing block 1 to block 2, block 2 to block 3, and block 3 to block 4.

#### Improvement with post-learning sleep

Our final questions of interest were: 1) whether intervening sleep between sessions improved choices from positive EV decks combined (C and D) and if so, 2) whether improvement was restricted to a specific deck? To calculate improvement the proportion of draws per 200 trials was calculated for session one and session two in both the combined positive EV decks (C and D) and each deck separately. The dependent variable was percent change (session 2 proportion – session 1 proportion). A one-way ANOVA was performed with condition (sleep, wake, sleep/wake) as the independent variable and percent change in positive EV decks (C and D) as the dependent variable. Further, a two-way ANOVA with condition (sleep, wake, sleep/wake) and deck (A, B, C, D) as the independent variables, and percent change as dependent variable was carried out.

## Results

### Initial Learning and Sleep Related Improvement

#### Session One: Initial Learning

**Figure 2:**
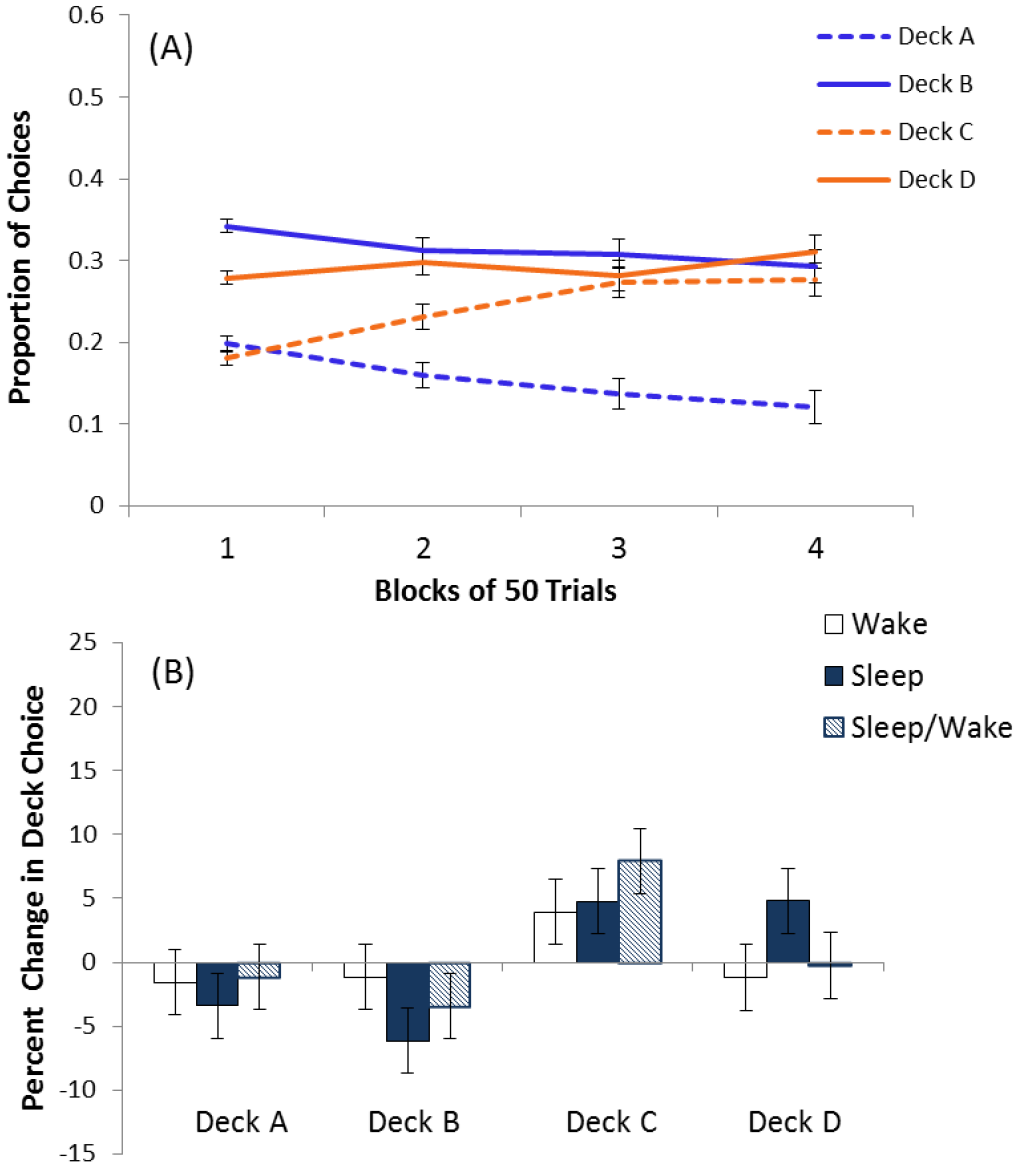
Session one deck choice and the subsequent improvement following a period of sleep and/or wake. (A) Session 1 proportion (± SEM) of draws from each deck (A, B, C, D) in 4 blocks of 50 trials for all participants combined over condition (N = 92). Solid lines represent low-frequency penalty decks and dashed lines represent high-frequency penalty decks; blue lines represent negative expected value (EV) decks and orange lines represent positive EV decks. In block 1 participants choose significantly more from deck B followed by deck D and least from decks A and C. In block 4 participants choose similarly from deck B, C, and D, each with significantly more choices than deck A. Supported by a significant block x deck interaction in a three-way analysis of variance (ANOVA) of block x deck x condition, followed by significant simple effects of decks at block 1 and block 4, followed by significant Tukey tests for pairwise comparisons. (B) Percent change (± SEM) in deck choice from session 1 to session 2 in wake (n = 26), sleep (n = 33) and sleep/wake control (n = 33) conditions. Percent change in Deck C was significantly different from percent change in Deck B in ANOVA of condition by deck; there were no significant effects of condition.

Averaging across conditions, in the first block (trials 1–50), deck B was most preferred and decks B and D were preferred over deck A and C (Fig. 2A). Over trials, preference for deck C increased and preference for deck A and B decreased (Fig. 2A). The block x deck x condition ANOVA revealed a significant block x deck interaction (F[6.5, 767.5]  = 10.26, p<0.01). All other main effects and interactions were not significant. Tests of simple effects of deck at block 1 revealed a significant effect (F[3, 364]  = 81.55, p<0.01) with Tukey tests showing deck B preferred over all decks, followed by deck D that was preferred over decks A and C. Tests of simple effects of deck at block 4 revealed a significant effect (F[3, 364]  = 19.1, p<0.01), with Tukey tests showing deck B, C and D preferred similarly and above deck A.

Across the four blocks, the repeated measures ANOVA revealed a significant change from deck A (F[3, 273]  = 26.7, p<0.01), deck B (F[2.4, 216.3]  = 3.3, p = 0.03) and deck C (F[1.9, 173.2]  = 9.5, p<0.01), and no significant change in deck D choice (F[2.1, 189.7]  = 2.1, p = 0.1). Choices from deck A significantly decreased from block 1 to block 2 (t[91]  = 4.3, p<0.01), and block 2 to block 3 (t[91]  = 2.6, p = 0.01). Choices from deck B significant reduced from block 1 to block 2 (t[91]  = 2.0, p = 0.048) and choices from deck C increased from block 1 to block 2 (t[91]  = −2.5, p = 0.01) and block 2 to block 3 (t[91]  = −2.7, p = 0.009) (Fig. 2A). No other paired t-tests were significant. These results demonstrate that participants showed evidence of learning, but still preferred deck B, C, and D in block 4, leaving room for improvement.

#### Performance Improvement Following Sleep

For the positive EV decks (C and D) combined, there was no significant effect of condition (F[2, 89]  = 1.48, *p* = 0.23). Figure 2B shows the percentage change in choices from each deck for each condition. Across all conditions, choices from deck C increased, choices from deck A and B decreased and choices from deck D stayed the same. We found no evidence that intervening sleep enhanced this improvement, evidenced by a non-significant deck x condition interaction (F[6, 356]  = 1.0, p = 0.41).

Close inspection of the data revealed that initial learning was driven by only a small proportion of individuals. A subset (approximately 1/3) of individuals improved, where the remaining participants had no improvement in session one. Considering it is well documented that that the benefits of sleep are largely reliant on achieving pre-sleep learning [19], [27], [28], [29], it is not surprising we were unable to find evidence for sleep-dependent improvement. Researchers often use performance level at the end of training to isolate those who showed evidence of learning [27], [35], [36], [22]. For this reason, we categorized individuals into learners and non-learners to determine whether improvement is localized to those who achieved some insight into the task prior to sleep.

### Learners and Non-learners: Categorization and Sleep-related Improvement

#### Session One: Categorization of Learners and Non-learners

**Figure 3:**
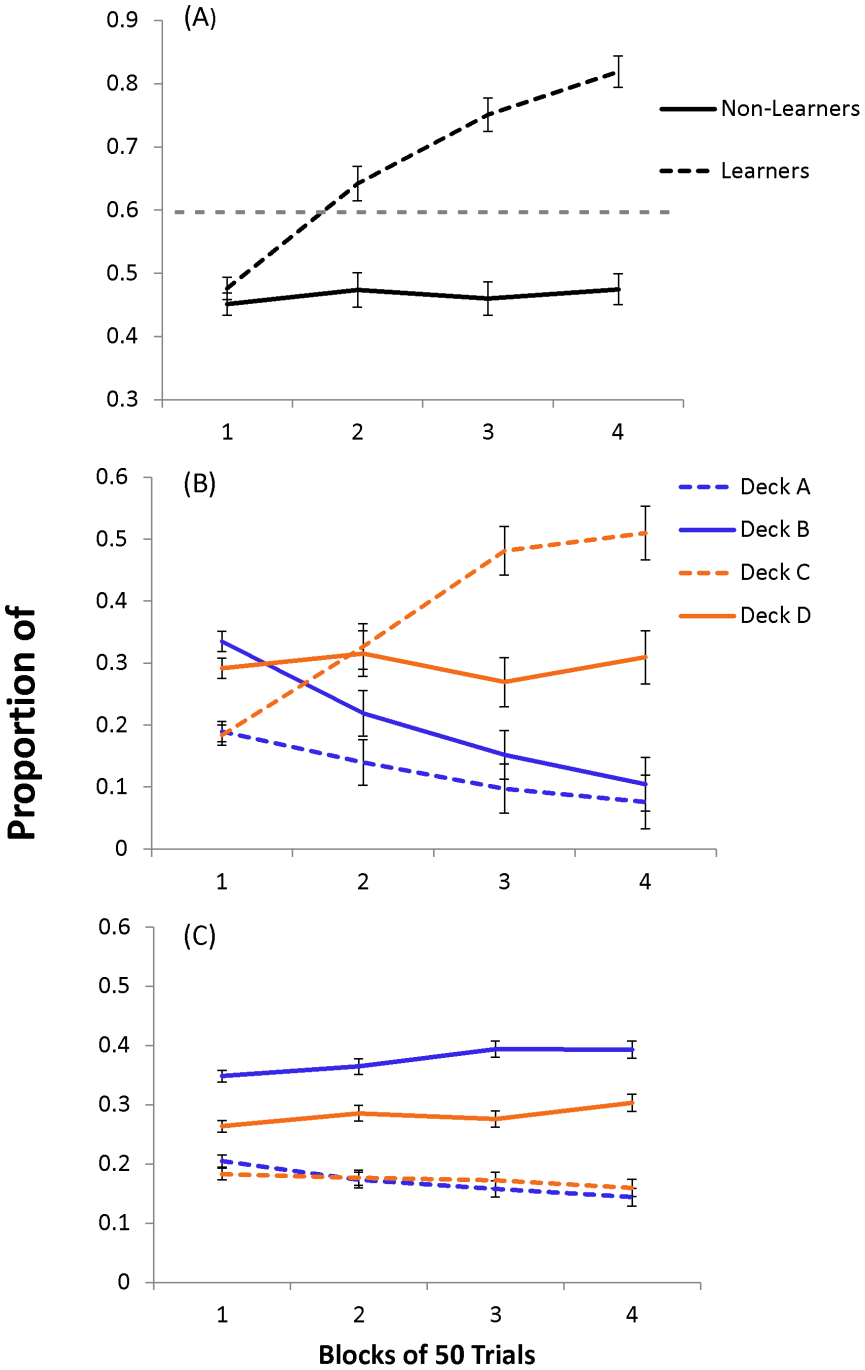
Proportion of deck choices from those categorized as learners and non-learners during session one. Session 1 proportion (± SEM) of draws in blocks of 50 trials from (A) positive expected value (EV) combined (C and D) for learners (n = 30) and non-learners (n = 62) as well as each individual deck (A, B, C, D) in (B) Learners and (C) Non-learners separately. All Learners reached 60% choices from good decks in the last 100 trials, the cut-off criterion is represented by the grey dotted line (A). Learners significantly improved choices from positive EV decks from block 1 to block 2, block 2 to block 3 and block 3 to block 4, with no significant improvement in the Non-Learners. Supported by a significant group by block interaction in a two-way analysis of variance, followed by a significant simple effects of block in Learners and significant Tukey tests of multiple comparison. The simple effects of block in Non-learners was non-significant. Learners appeared to reduce preference for deck B and increase choices from deck C (B), while Non-learners did not appear to reduce deck B preference (C). Solid lines represent low-frequency penalty decks and dashed lines represent high-frequency penalty decks; blue lines represent negative EV decks and orange lines represent positive EV decks.

Individuals were categorized into learners (n = 30) and non-learners (n = 62) based on their performance in the last half of session one (total draws chosen from deck C and D combined in trials 101–200). Using individual observations, and a previously established criterion [27], [35] we categorized learners and non-learners as those who reached equal or more than 60% and less than 60% choices from combined good decks (decks C and D), respectively. A significant two-way block (1, 2, 3, 4) x group (learners, non-learners) ANOVA confirmed group differences (F[2.7, 239.9]  = 37.6, p<0.001). In the learners, a repeated measures ANOVA revealed that choices from good decks significantly improved (F[2.4, 68.9]  = 36.1, p<0.001) with no significant improvement in non-learners (F[2.6, 160.7]  = 1.1, p = 0.34). Within learners paired t-tests revealed choices from good decks significantly increased from block 1 to block 2 (t[29]  = −3.9, p<0.001), block 2 to block 3 (t[29]  = −3.8, p<0.001), and block 3 to block 4 (t[29]  = −2.7, p<0.05). Furthermore, Figure 3B suggests that learners decreased preferences for deck B, choosing predominately from good decks C and D in the last 50 trials. Figure 3C suggests that non-learners prefer deck B and D throughout the full 200 trials.

The percentage of individuals classified as learners was slightly larger in the wake (38%; N = 10), compared to the sleep (30%; N = 10) and sleep/wake (30%; N = 10) groups. To ensure improvement was equal between the three conditions we ran a block (1, 2, 3, 4) x condition (sleep, wake, sleep/wake) ANOVA with proportion of choices from positive EV decks as the dependent variable. As expected, the learners had a significant main effect of block (F[2.3, 63.0]  = 35.5, p<0.001), and non-significant main effect of condition (F[2], [27]  = 1.0, p = 0.37) and block x condition interaction (F[4.7, 63.0]  = 0.77, p = 0.57). These results show that improvement in session one was consistent among conditions.

#### Learners and Non-learners: Performance Improvement Following Sleep

Our final question was whether sleep-dependent improvement was exclusive to those with initial learning. A two-way ANOVA was performed with group (learners, non-learners) and condition (sleep, wake, sleep/wake) as the independent variables and percent change in the positive EV decks (C and D) as the dependent variable. The same simple effects ANOVA as the original analysis was performed for learners and non-learners separately. Pairwise comparisons were made with Tukey tests. To investigate improvement across individual decks, a three-way ANOVA was performed with group (learners, non-learners), deck (A,B,C,D), and condition (sleep, wake, sleep/wake) as independent variables and percent change as a dependent variable. The same two-way condition x deck ANOVA as the original analysis was performed for learners and non-learners separately. This was followed by individual simple effects ANOVA of condition (sleep, wake, sleep/wake) for each deck, and Tukey tests for multiple comparisons.

**Figure 4:**
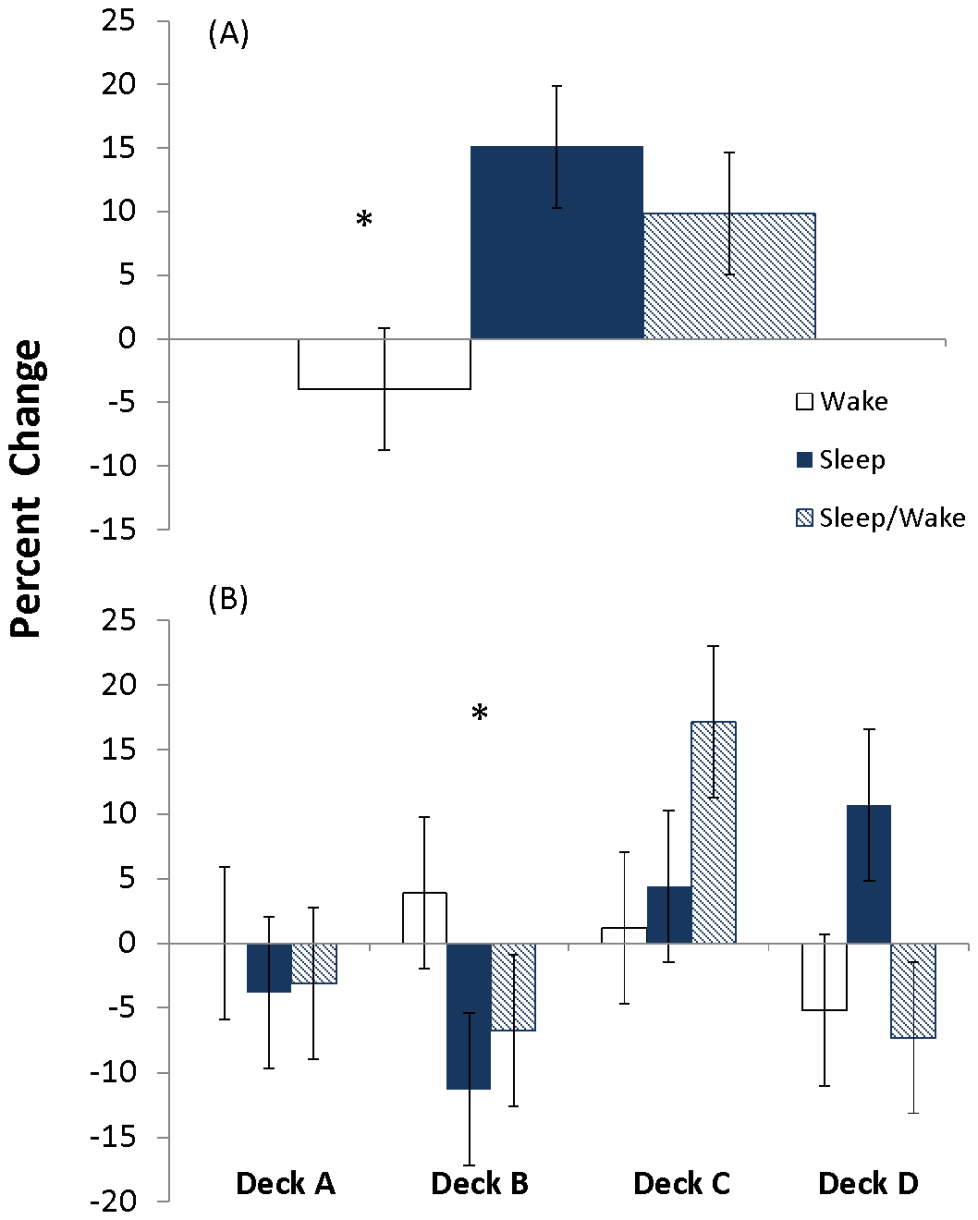
Improvement in deck choice following sleep and/or wake in learners. Percent change (± SEM) from session 1 to session 2 in wake (n = 10), sleep (n = 10) and sleep/wake control (n = 10) conditions in draws from (A) good overall expected value decks (C and D) and (B) individual decks (A, B, C, and D) of individuals who were categorized as learners in session 1. Improvement is reflected by a negative percent change in deck A and B and positive percent change in deck C and D. * Significant improvement (p<0.05) for both sleep groups (sleep and control) compared to wake by one-way analysis of variance (ANOVA) followed by Tukey tests (A) and by two-way ANOVA revealing a significant (p<0.05) interaction followed by significant simple effects of group for deck B (p = 0.02) followed by Tukey tests (B).

**Figure 5:**
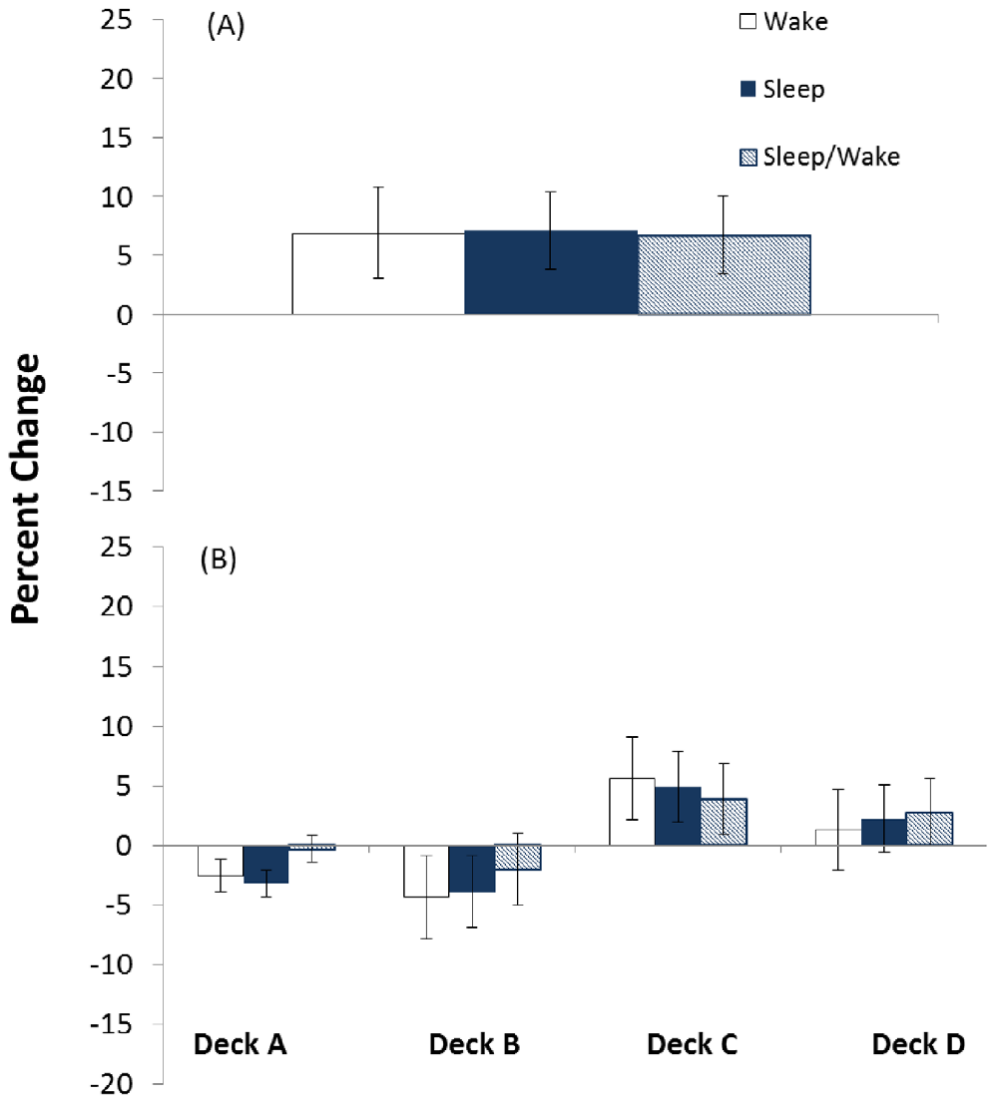
Improvement in deck choice following sleep and/or wake in non-learners. Percent change (± SEM) from session 1 to session 2 in wake (n = 16), sleep (n = 23) and sleep/wake control (n = 23) conditions in draws from (A) good overall expected value decks (C and D) and (B) individual decks (A, B, C, and D) of individuals who were categorized as non-learners in session 1. Improvement is reflected by a negative percent change in deck A and B and positive percent change in deck C and D. The sleep groups (sleep and control) did not significantly change compared to the wake group, as evidenced by a non-significant one-way analysis of variance (ANOVA) (A) and two-way ANOVA (B).

Within the learners, the conditions that had intervening sleep (sleep and sleep/wake control) had a significantly larger improvement in choice from positive EV decks compared to the wake condition (Fig. 4A). The two-way ANOVA revealed a significant group (learners, non-learners) x condition (sleep, wake, sleep/wake) interaction (F[2,92]  = 2.7, p = 0.04). A one-way ANOVA in the learners revealed a significant main effect of condition (F[2], [27]  = 4.98, p = 0.014). Tukey tests revealed the sleep (p = 0.005) and sleep/wake control (p = 0.03) conditions exhibited a larger percent change in positive EV decks than the wake condition. In the non-learners, a one-way ANOVA of condition revealed a non-significant effect (F[2,59]  = 0.004, p = 0.996). Results revealed a similar percent change in choices from positive EV decks (C and D) in the sleep, sleep/wake control and wake groups (Fig. 5A.).

Within the learners, the conditions that had intervening sleep (sleep and sleep/wake control) had a significantly larger reduction in choices from deck B compared to the wake condition (Fig. 4B). The group x condition x deck ANOVA revealed a significant three-way interaction (F[6,344]  = 2.7, p = 0.014). In the learners, a deck x condition ANOVA revealed a significant interaction (F[6,108]  = 2.26, p = 0.043). Test of simple effects of condition were significant for deck B (F[2], [27]  = 7.61, p = 0.02). The sleep (p<0.01) and sleep/wake control (p = 0.015) condition decreased choices towards deck B significantly more than the wake condition. There was a non-significant effect of condition for deck A (F[2], [27]  = 0.74, p = 0.48), deck C (F[2], [27]  = 0.96, p = 0.4) and deck D (F[2], [27]  = 1.93, p = 0.16).

In the non-learners, there was no evidence that intervening sleep enhancement improvement, as evidenced by a non-significant deck x condition ANOVA (F[6, 236]  = 0.16, p = 0.99). The wake, sleep, and sleep/wake control condition showed similar respective percent changes (± SEM), reducing choices from bad decks A and B and increasing choices toward good decks C and D (Fig. 5B).

## Discussion

This study reveals the first clear evidence that post-learning sleep facilitates decision-making of the IGT. Individuals with initial learning had larger post-learning improvement following a period of sleep compared to a period of wake (Fig. 4A). More specifically, we are the first to demonstrate that sleep enhances performance via reduction in deck B (Fig. 4B) and increase in positive EV decks combined (Fig. 4A).

As hypothesized, initial learning required reduction in choices from initially preferred bad deck B, and increase toward positive EV deck C and/or D (Fig. 2A). Individuals improved in session one (Fig. 2A) and showed further improvement during session two (Fig. 2B), however, we found no initial evidence to support the idea that intervening sleep enhanced this process (Fig. 2B). Close inspection of the data revealed that initial learning in session one was driven by about one-third of participants. These learners decreased choice from deck B and increased choice from deck C, gaining preference for positive EV decks at the end of session one (Fig. 3B). The remaining non-learners preferred deck B throughout the 200-trials of session one (Fig. 3C). Within the learners there was a significant decrease in deck B (Fig. 4B) and increase in positive EV decks C and D following a period of intervening sleep compared to a period of intervening wake (Fig. 4A).

Our results build on previous work by Abe et al. [26] by ruling out potential influences of time-of-day or hours of prior wakefulness on improvement. The sleep/wake control was administered session two at the same time as our wake condition (21:00–22:00) and showed similar improvement to the sleep group who was administered session two in the morning (09:00–10:00) (Fig. 1). We also found no evidence that time-of-day (morning vs. evening) influenced initial performance on the task during session one and subjective sleepiness scores did not significantly change between sessions or conditions. These results support the conclusion that improvement was due to intervening sleep rather than time-of-day or hours of prior wakefulness.

These results are the first to show strong evidence that post-learning sleep can improve performance on a task that was designed to mimic real life decision-making. In the past, sleep has been shown to enhance performance on many types of learning tasks, including motor procedural [30], neutral declarative [37], emotional declarative [23] and complex cognitive tasks [19], [20], [22]. The IGT is unique from these tasks, and referred to as a cognitive-emotional task that integrates emotional response to decks in the vmPFC [38], [39], [40]. This is the first evidence that sleep can help promote learning of this nature. In addition, our results show that sleep may specifically enhance learning of deck B. Deck B has a unique design. It is initially preferred, however, individuals must integrate the large infrequent $1250 loss into their value estimate to discover it has a negative EV. Numerous studies have revealed that IGT learning is guided by experienced and anticipated galvanic skin response toward bad decks [38], [39], [40]. Thus, experiencing negative emotions to deck B may be imperative for IGT learning and subsequently enhanced with sleep. There has yet to be a study to investigate the online neural mechanisms of this shift.

These results support the sleep deprivation literature that suggests cognitive processes of IGT decision-making are intricately connected with sleep behaviour. A recent study by Venkatraman et al. [45] found that IGT impairment following sleep deprivation was due to a generalized dampened activity in the vmPFC, and reduced activity in insula following a loss. The authors suggest that sleep deprivation may impair the ability to learn from previous negative experiences. Likewise, learning in the IGT, is marked by heightened activity in the vmPFC [7] and insula [8]. Thus, one potential hypothesis for our results is that the heightened activity of the vmPFC and insula that occurs while integrating loss information into deck evaluations, may be subsequently processed and further enhanced during a period of intervening sleep. Considering what is currently known in the IGT and sleep literature, we speculate that rapid eye movement (REM) sleep may be specifically involved in this process.

REM sleep has been shown to enhance learning of complex cognitive tasks [19], [20], [21], [22], as well as memory for stimuli that evoke negative emotions [23], [24]. Additionally, several areas that are active during online learning of the IGT, including the ventral striatum, amygdala, vmPFC and hippocampus [7], [8], [9], [13], have heightened activity during REM sleep, compared to activity in other stages of sleep and quiet wakefulness [41]. It was recently suggested by the Reward Activation Model that the emotionally driven pathways active during wake are reactivated during REM sleep, and this reactivation contributes to memory enhancement [41]. Thus, one potential explanation for our results is that REM strengthens previously acquired learning by reactivating neural pathways that were active during online learning. Furthermore, given the general role of REM in processing negative emotions [23], [24], post-learning REM may be particularly beneficial for processing negative emotions to deck B loss.

Another potential hypothesis for our results is that post-learning sleep may consolidate declarative memory for deck representations through slow-wave sleep (SWS). SWS strengthens memories that rely on hippocampal activity through activation of hippocampal-cortical connections [37], [42]. It is possible that learners gained an explicit knowledge of deck B during session one that was further enhanced via SWS. However, due to the relatively little understanding of the neural mechanisms of individual deck choice, and the neural processes of sleep, we can only speculate about the mechanisms for consolidation. Future research to investigate these hypotheses would provide great insight into the role of post-learning sleep on decision-making processes.

In addition to our ‘deck B sleep effect’ choices trended toward deck D in the sleep condition, and deck C in the sleep/wake control condition (Fig. 4B). These differences failed to reach significance and we found no evidence that sleep improved performance on C or D separately. Currently it is unclear what drives individuals toward deck C, deck D or a combination of deck C and D and how individual strategies might evolve. Future studies could use imaging techniques to investigate the underlying deck characteristics and neural mechanisms that contribute to online and offline processing of deck B and positive EV decks.

One surprising observation was that initial learning in session one was driven by approximately 33% of participants. A likely explanation is that our version was designed for difficultly. We also refrained from giving participants a “hint” that some decks were better than others, which has been shown to significantly slow learning [5]. It could also be argued that our relatively young age (mean age  = 20.7± SEM 0.2) [43], and large percentage of female participants in the study (79%) [34] contributed to this effect. However, our data revealed no evidence that age or gender influenced performance. Currently, it is unclear what might distinguish a learner from non-learner. We suggest that categorizing learners and non-learners may be a useful tool for future studies. Future work could investigate whether learners and non-learners have distinct cognitive and neural processes that influence both online and offline improvement on the task.

### Limitations and Outstanding Questions

One potential limitation in our study is that we have not fully controlled for all circadian factors that might influence IGT performance. Although we have controlled for differences in time of re-testing and hours of prior wakefulness between our sleep and wake conditions, factors such as the timing of the sleep and wake periods, and the timing of the sleep period that followed learning, were not fully controlled. To fully understand the role of sleep in processing IGT learning, outstanding questions to address are: 1) does a sleep period during the day and wake period during the evening elicit similar sleep-dependent learning effects, and 2) does the timing of initial learning, and/or the length of time between learning and sleep influence sleep-dependent enhancement? Future research should seek to understand how sleep related processes and processes that modulate circadian rhythms interact and influence IGT choice.

A potential factor that may limit the generalizability of our results is the use of virtual money, rather than having participants compensated with real money based on their performance. Recent evidence has found that sleep-dependent consolidation is enhanced when the belief of future reward expectancy is introduced prior to sleep. That is, sleep-dependent learning is mediated by the relevance that is assigned to the task prior to sleep [44], [45]. Thus, it is possible that sleep-dependent improvement in the IGT is more pronounced when decisions have high intrinsic motivation and mimic real-life scenarios.

## Conclusion

In summary, we are the first to provide evidence that post-learning sleep can enhance performance on a task designed to mimic real life decision-making. These findings provide new insights into IGT learning, and support the hypothesis that ‘sleeping on it’ facilitates decision-making. These findings have important implications for the role of sleep in processing decision-making experiences in a wide variety of populations. Future work should be aimed at identifying the underlying cognitive and emotional processes and corresponding sleep mechanisms that help facilitate this unique process.

## References

[1] Bechara A, Damasio AR, Damasio H, Anderson SW (1994) Insensitivity to future consequences following damage to prefrontal cortex. Cognition 50: 7–15 Doi: 10.1016/0010-0277(94)90018-3 8039375 10.1016/0010-0277(94)90018-3

[2] Chiu Y, Lin C, Huang J, Lin S, Lee P, et al (2008) Immediate gain is a long-term loss: Are there foresighted decision makers in the iowa gambling task? Behav Brain Funct 4: 13 Doi: 10.1186/1744-9081-4-13 18353176 10.1186/1744-9081-4-13 PMC2324107

[3] Lin C, Chiu Y, Lee P, Hsieh J (2007) Is deck B a disadvantageous deck in the iowagambling task? Behav Brain Funct 3: 16 Doi: 10.1186/1744-9081-3-16 17362508 10.1186/1744-9081-3-16 PMC1839101

[4] Singh V, Khan A (2009) Heterogeneity in choices on iowa gambling task: Preference for infrequent-high magnitude punishment. Mind and Society 8: 43–57 Doi: 10.1007/s11299-008-0050-1

[5] Fernie G, Tunney RJ (2006) Some decks are better than others: The effect of reinforcer type and task instructions on learning in the iowa gambling task. Brain Cogn 60: 94–102 Doi: 10.1016/j.bandc.2005.09.011 16271818 10.1016/j.bandc.2005.09.011

[6] Wasserman JI, Barry RJ, Bradford L, Delva NJ, Beninger RJ (2012) Probabilistic classification and gambling in patients with schizophrenia receiving medication: Comparison of risperidone, olanzapine, clozapine, and typical antipsychotics. Psychopharmacology 222 (1): 173–183 Doi: 10.1007/s00213-011-2634-4 22237855 10.1007/s00213-011-2634-4

[7] Ernst M, Bolla K, Mouratidis M, Contoreggi C, Matochik JA, et al (2002) Decision-making in a risk-taking task: A PET study. Neuropsychopharmacology 26 (5): 682–691 Doi: 10.1016/S0893-133X(01)00414-6 11927193 10.1016/S0893-133X(01)00414-6

[8] Lawrence NS, Jollant F, O'Daly O, Zelaya F, Phillips MJ (2008) Distinct roles of prefrontal cortical subregions in the Iowa Gambling Task. Cereb Cortex 19: 1134–1143 Doi: 10.1093/cercor/bhn154 18787233 10.1093/cercor/bhn154

[9] Linnet J, Moller A, Peterson E, Gjedde A, Doudet D (2010) Dopamine release in the ventral striatum during Iowa Gambling Task performance is associated with increased excitement levels in pathological gamblers. Addiction 106: 383–390. 20883460 10.1111/j.1360-0443.2010.03126.x

[10] Bechara A, Damasio H, Damasio AR, Lee GP (1999) Different contributions of the human amygdala and ventromedial prefrontal cortex to decision-making. Journal Neurosci 19 (13): 5473–5481. 10.1523/JNEUROSCI.19-13-05473.1999 PMC6782338 10377356

[11] Lin CH, Chiu YC, Cheng CM, Hsieh JC (2008) Brain maps of Iowa Gambling Task. BMC Neurs: 9 Doi: 10.1186/1471-2202-9-72 10.1186/1471-2202-9-72 PMC2518922 18655719

[12] Li X, Lu Z, D'Argembeau A, Ng M, Bechara A (2010) The iowa gambling task in fMRI images. Hum Brain Mapp 31: 410–423 Doi: 10.1002/hbm.20875 19777556 10.1002/hbm.20875 PMC2826566

[13] Gupta R, Duff MC, Denburg NL, Cohen NJ, Bechara A, et al (2009) Declarative memory is critical for sustained advantageous complex decision-making. Neuropsychologia 47: 1686–1693 Doi: 10.1016/j.neuropsychologia.2009.02.007 19397863 10.1016/j.neuropsychologia.2009.02.007 PMC2697903

[14] Venkatraman V, Chuah YM, Huettal SA, Chee MW (2007) Sleep deprivation elevates expectations of gains and attenuates response to losses following risky decisions. Sleep 30: 603–609 Doi: 10.3109/07420528.2011.635230 17552375 10.1093/sleep/30.5.603

[15] Killgore DS, Grugle NL, Balkin TJ (2012) Gambling when sleep deprived: Don't bet on stimulants. Chronobiol Int 29: 43–54 Doi: 10.3109/07420528.2011.635230 22217100 10.3109/07420528.2011.635230

[16] Rasch B, Born J (2013) About sleep's role in memory. Physiology Review 93: 681–766 Doi: 10.1152/physrev.00032.2012 10.1152/physrev.00032.2012 PMC3768102 23589831

[17] Smith C (1995) Sleep states and memory processes. Behav Brain Res 69: 137–145 Doi: 10.1016/0166-4328(95)00024-N 7546305 10.1016/0166-4328(95)00024-n

[18] Smith CT, Peters KR (2011) Sleep, memory and molecular neurobiology. Handb Clin Neurol 98: 259–272 Doi: 10.1016/B978-0-444-52006-7.00017-4 21056192 10.1016/B978-0-444-52006-7.00017-4

[19] Djonlagic I, Rosenfeld A, Shohamy D, Myers C, Gluck M, et al (2009) Sleep enhances category learning. Learn Mem 16: 751–755 Doi: 10.1101/lm.1634509 19926780 10.1101/lm.1634509 PMC2788212

[20] Ellenbogen JM, Hu PT, Payne JD, Titone D, Walker MP (2007) Human relational memory requires time and sleep. Proc Natl Acad Sci USA 104 (18): 7723–7728 Doi: 10.1073/pnas.0700094104 17449637 10.1073/pnas.0700094104 PMC1863467

[21] Tse D, Langston RF, Kakeyama M, Bethus I, Spooner PA, et al (2007) Schemas and memory consolidation. Science 316 (5821): 76–82 Doi: 10.1126/science.1135935 17412951 10.1126/science.1135935

[22] Yordanova J, Kolev V, Verleger R, Bataghva Z, Born J, et al (2008) Shifting from implicit to explicit knowledge: Different roles of early and late night sleep. Learn Mem 15: 508–515 Doi: 10.1101/lm.897908 18626095 10.1101/lm.897908 PMC2505318

[23] Baran B, Pace-Schott EF, Ericson C, Spencer RMC (2012) Processing of emotional reactivity and emotional memory over sleep. Journal Neurosci 32 (3): 1035–1042 Doi: 10.1523/JNEUROSCI.2532-11.2012 10.1523/JNEUROSCI.2532-11.2012 PMC3548452 22262901

[24] Nishida M, Pearsall J, Buckner RL, Walker MP (2009) REM sleep, prefrontal theta, and the consolidation of human emotional memory. Cereb Cortex 19: 1158–1166 Doi: 10.1093/cercor/bhn155 18832332 10.1093/cercor/bhn155 PMC2665156

[25] Pace-Schott EF, Nave G, Morgan A, Spencer RMC (2012) Sleep-dependent modulation of affectively guided decision-making. J Sleep Res 21: 30–39 Doi: 10.1111/j.1365-2869.2011.00921 21535281 10.1111/j.1365-2869.2011.00921.x PMC3150608

[26] Abe T, Inoue Y, Komada Y, Hori T (2012) Effect of post-learning sleep versus wakefulness on advantageous decision-making: A preliminary study. Sleep Biol Rhythms 10: 72–74 Doi: 10.1111/j.1479-8425.2011.00509.x

[27] Fogel SM, Smith CT, Beninger RJ (2009) Evidence for 2-stage models of sleep and memory: Learning-dependent changes in spindles and theta in rats. Brain Res Bull 79 (6): 445–451 Doi: 10.1016/j.brainresbull.2009.03.002 19559345 10.1016/j.brainresbull.2009.03.002

[28] Peigneux P, Laureys S, Fuchs S, Destrebecqz A, Collette FA (2003) Learned material content and acquisition level modulate cerebral reactivation during posttraining rapid-eye-movement sleep. Neuroimage 20: 125–134 Doi: 10.1016/S1053-8119(03)00278-7 14527575 10.1016/s1053-8119(03)00278-7

[29] Wilhelm I, Metzkow-Meszaros M, Knapp S, Born J (2012) Sleep-dependent consolidation of procedural motor memories in children and adults: The pre-sleep level of performance matters. Dev Sci 15 (4): 506–515 Doi: 10.1111/j.1467-7687.2012.01146.x 22709400 10.1111/j.1467-7687.2012.01146.x

[30] Peters KR, Smith V, Smith CT (2007) Changes in sleep architecture following motor learning depend on initial skill level. J Cogn Neurosci 19: 817–829 Doi: 10.1162/jocn.2007.19.5.817 17488206 10.1162/jocn.2007.19.5.817

[31] Preston SD, Buchanan TW, Stansfield RB, Bechara A (2007) Effects of anticipatory stress on decision making in a gambling task. Behav Neurosci 121 (2): 257–263 Doi: 110.1037/0735-7044.121.2.257 17469915 10.1037/0735-7044.121.2.257

[32] Johns MW (1991) A new method for measuring daytime sleepiness: The Epworth sleepiness scale. Sleep 14: 540–545. 1798888 10.1093/sleep/14.6.540

[33] Seeley CJ, Cashaback JGA, Smith CT Beninger RJ (2014) Altering the shape of distributions affects the decision-making in a modified Iowa Gambling Task. J Behav Decis Mak 27: 170–178 Doi: 10.1002/bdm.1795

[34] Overman WH, Pierce A (2013) Iowa Gambling Task with non-clinical participants: Effects of using real + virtual cards and additional trials. Front Psychol 4: 935 Doi: 10.3389/fpsyg.2013.00935 24376431 10.3389/fpsyg.2013.00935 PMC3859904

[35] Fogel SM, Smith CT, Beninger RJ (2010) Too much of a good thing? Elevated baseline sleep spindles predict poor avoidance performance in rats. Brain Research 1319: 112–117 Doi: 10.1016/j.brainres.2010.01.026 20083090 10.1016/j.brainres.2010.01.026

[36] Smith C, Wong PTP (2001) Paradoxical sleep increases predict successful learning in a complex operant task. Behav Neurosci 105: 282–288 Doi: 10.1037/0735-7044.105.2.282 10.1037//0735-7044.105.2.282 2043274

[37] Diekelmann S, Wilhelm I, Born J (2009) The whats and whens of sleep-dependent memory consolidation. Sleep Med Rev 13: 309–321 Doi: 10.1016/j.smrv.2008.08.002 19251443 10.1016/j.smrv.2008.08.002

[38] Bechara A, Tranel D, Damasio H, Damasio AR (1996) Failure to response autonomically to anticipated future outcomes following damage to prefrontal cortex. Cereb Cortex 6: 215–225 Doi: 10.1093/cercor/6.2.215 8670652 10.1093/cercor/6.2.215

[39] Crone EA, Somsen RJ, Van Beek B, Van der Molen MW (2004) Heart rate and skin conductance analysis of antecedents and consequences of decision making. Psychophysiology 41: 531–540 Doi: 10.1111/j.1469-8986.2004.00197.x 15189476 10.1111/j.1469-8986.2004.00197.x

[40] Guillaime S, Jollant F, Jaussent I, Lawrence N, Malafosse A, et al (2009) Somatic markers and explicit knowledge are both involved in decision-making. Neuropsychologia 47 (10): 2120–2124 Doi: 10.1016/j.neuropsychologia.2009.04.003 19427005 10.1016/j.neuropsychologia.2009.04.003

[41] Perogamvros L, Schwartz S (2012) The roles of the reward system in sleep and dreaming. Neurosci Biobehav Rev 36: 1934–1951 Doi: 10.1016/j.neubiorev.2012. 05.010 22669078 10.1016/j.neubiorev.2012.05.010

[42] Schabus M, Gruber G, Parapatics S, Sauter C, Klosch G, et al (2004) Sleep spindles and their significance for declarative memory consolidation. Sleep 27: 1479–1485. 15683137 10.1093/sleep/27.7.1479

[43] Cauffman E, Shulman EP, Steinberg L, Claus E, Banich MT, et al (2010) Age differences in affective decision making as indexed by performance on the Iowa Gambling Task. Dev Psychol 46: 193–207 Doi: 10.1037/a0016128 20053017 10.1037/a0016128

[44] Fischer S, Born J (2009) Anticipated reward enhances offline learning during sleep. J Exp Psychol Learn Mem Cogn 35: 1586–1593 Doi: 10.1037/a0017256 19857029 10.1037/a0017256

[45] Wilhelm I, Diekelmann S, Molzow I, Ayoub A, Molle M, et al (2011) Sleep selectively enhances memory expected to be of future relevance. Journal Neurosci 31: 1563–1569 Doi: 10.1523/JNEUROSCI.3575-10.2011 10.1523/JNEUROSCI.3575-10.2011 PMC6623736 21289163

